# Supplementary material for: Mining the equine gut metagenome: poorly-characterized taxa associated with cardiovascular fitness in endurance athletes
Source: Commun Biol. 2022 Oct 3;5:1032. doi: 10.1038/s42003-022-03977-7 (PMC9529974; doi:10.1038/s42003-022-03977-7)

SUPPLEMENTARY NOTE

Physiological response to endurance exercise in highly trained athletes

Since endurance racing causes great physiological stress on the body, not surprisingly, all horses showed above-average total bilirubin, creatine, creatine kinase (CK), aspartate transaminase, and serum concentrations of amyloid A after the race (Supplementary Data 17). The elevated creatine level and CK activity likely reflected higher membrane permeability associated with muscle protein leakage to the blood and severe inflammation signaling [1]. Similarly, substantial muscle turnover increased the production of creatine in athletes [2].

The study of the adaptive metabolism response to endurance exercise through metabolomics pinpointed that the concentration of lactate (a proxy for glycolytic stress and disturbances in cellular homeostasis [3]) was significantly increased after the race, as well as the levels of fatty acids from lipoproteins and of specific amino acids, namely alanine, branched amino acids such as leucine, valine and *iso*-valerate, glutamate, glutamine and aromatic amino acids such as tyrosine and phenylalanine (Supplementary Data 16). Moreover, ketone bodies increased slightly after the race (*i.e.,* acetoacetate and acetate; Supplementary Data16). Accordingly, the non-esterified fatty acids and the β-hydroxybutyric concentrations showed similar patterns, indicating increased lipolysis. The elevated level of lipid utilization during endurance exercise was confirmed by the greater mitochondrial β-oxidation activity, reflected by increased blood acylcarnitine concentrations (from 6.06 ± 1.85 to 42.38 ± 13.02 µmol/L; Supplementary Data 18).

The gut microbiome contains vast and individual-specific genetic content.

We sought to determine the frequency of occurrence of each gene on a sample-by-sample basis. The gene catalog contained 295,948 (1.17%) singletons. On average, 2.5% of the genes in each sample were singletons (standard deviation of 30.58%). Moreover, the gene abundances and the percentages of the genes shared among samples indicated that most identified genes had lower abundance/prevalence within the individual samples (Supplementary Figure 1e).

Supplementary description of the microbial gut catalog taxonomic assignation

Taxonomic inference relying on the eggNOG database yielded an annotation for 16,795,097 genes, but only 3,205,480 (12%) could be classified down to the genus level. Moreover, significant discrepancies were found while attempting to cross-validate this orthology-based annotation with available 16S rRNA sequences (Supplementary Data 10-11). Indeed, the most prominent genera were not classified on that basis. For instance, orthology-based inference performed poorly at classifying sequences of the most abundant genera such as *Treponema*, *Clostridium* XIVa, *Prevotella, Ruminococcus,* and *Fibrobacter*, which altogether accounted for most of the gut microbiota composition (56% of 16S rRNA reads).

The metagenome-assembled genomes (MAGs) slightly capture the complexity of the natural population

To uncover whether the recovery of MAGs were appropriate proxies for the whole microbial communities or whether they only captured the most abundant microorganisms, MAGs were constructed from the metagenomic sequencing obtained from the 11 samples described above using the same metagenomic ATLAS pipeline v. 2.4.4 [4].
The mean percent completeness and contamination of the 372 non-redundant MAGs were 83% ± 12% and 1.7% ± 2.05%, respectively (Supplementary Figure 2a-c). They were then classified into taxa using the Genome Taxonomy Database Toolkit (GTDB-Tk) [5]. Completeness was not significantly correlated with genome size ($r^2 = 0.17$, $p = 0.55$; Supplementary Figure 2d). All

MAGs together totaled 53,149 contigs (34% of total assembly) with an average assembly N50 value of 4.5 Kb (Supplementary Figure 2f) and CG content of 44.49% (Supplementary Data 8). Genome sizes ranged from 0.42 to 88.97 Mb (Supplementary Figure 2e). The two highest contig NG50s were about 0.233 to 0.459 Mb. MAGs missed 94.5% and 97.7% of the catalog's population core and variable genes.

MAGs did not capture the taxonomic level's complexity and diversity observed in the gene catalog nor mirrored the 16S rRNA gene amplification data (Supplementary Figure 6). As specified above, the MAG repertoire brought read classification rates up to 30% of the data, suggesting that the remaining reads were likely from low-abundance bacterial and archaeal species, difficult-to-assemble genomes, and the fungal, protozoan, and viral genomes. That said, our results provide a reference guide for the horse community. We compared our 372 MAGs to the publicly available MAGs in horses to date [6,7]. None of our MAGs showed complete identity to any other publicly available MAG in the horse, although three MAGs were more than 99% similar to another MAG published in the horse, namely MAG1 and MAG241, and MAG322.

We then mapped our MAG proteins to the CAZymes database. A total of 103 active enzyme families were identified. The most abundant enzyme classes were the carbohydrate degrading GHs (n = 52) and GTs (n = 34, Supplementary Data 8). The phylogenetic tree based on genus level showed that most MAGs have GHs enzymes, a complex and widespread group of enzymes that degrade polysaccharides (Supplementary Figure 3). Bacteroidetes captured greater CAZyme diversity, as they showed an increased number of CAZYmes families, and these families contain more enzymes than others (Supplementary Data 8). A total of 7,186 KO was reported (Supplementary Data 8).

The association between cardiovascular fitness and gut metagenome revalidated by MAGs

Knowing that the 372 nearly-complete MAGs only represented a small fraction of the global metagenome community, we wanted to assess if they could at least provide critical data related to the ecological and genetic mechanisms responsible for the diversity patterns observed between individuals with divergent cardiovascular capacities. Consistent with the dominant phylotypes, samples from individuals with different cardiovascular fitness could be distinguished by MAGs composition. The ordination plot based on the β-diversity showed that samples from individuals with similar cardiovascular capacity tended to cluster closely (p = 0.005, R^2 = 0.1543, PerMANOVA of Bray-Curtis distances), suggesting that the 372 reliably captured the overall structure of the gut microbial community. Conversely, the α-diversity indices comparison indicated that all samples had similar levels of diversity (p = 0.9212 for Shannon and inverse Simpson, two-sided Wilcoxon rank-sum test) regardless of the cardiovascular capacity. Having found that MAG composition was segregated into two clusters, we established that this compositional dissimilarity did not have any functional ability, probably due to the poor annotations of metagenomic sequencing data at the species level (Supplementary Figure 6a-d).

We then focused on those MAGs and their functional capacities enriched between individuals with different cardiovascular fitness using the DESeq2 package. The abundance of 52 MAGs was statistically different between groups (adjusted $p < 0.05$ with DESeq2; Supplementary Figure 6c). Changes in MAGs community composition were most pronounced in individuals with improved cardiovascular fitness, increasing the relative abundance of Verrucomicrobia (*e.g., Akkermansia* spp.) and Bacteroidetes. On the other hand, they harbored a lower relative

abundance of Fibrobacteres and Spirochaetes. Nevertheless, overall CAZymes and KOs profiles were unchanged by group (Supplementary Figure 6d), raising awareness that our MAG collection likely underrepresented the whole metagenome and could not find distinct metabolic affinities between participants.

SUPPLEMENTARY REFERENCES

1. Capomaccio, S. *et al.* Microarray analysis after strenuous exercise in peripheral blood mononuclear cells of endurance horses. *Anim. Genet.* **41**, 166–175 (2010).
2. O'Donovan, C. M. *et al.* Distinct microbiome composition and metabolome exists across subgroups of elite Irish athletes. *J. Sci. Med. Sport* **23**, 63–68 (2020).
3. Hawley, J. A., Lundby, C., Cotter, J. D. & Burke, L. M. Maximizing Cellular Adaptation to Endurance Exercise in Skeletal Muscle. *Cell Metab.* **27**, 962–976 (2018).
4. Kieser, S., Brown, J., Zdobnov, E. M., Trajkovski, M. & McCue, L. A. ATLAS: A Snakemake workflow for assembly, annotation, and genomic binning of metagenome sequence data. *BMC Bioinformatics* **21**, 257 (2020).
5. Chaumeil, P. A., Mussig, A. J., Hugenholtz, P. & Parks, D. H. GTDB-Tk: A toolkit to classify genomes with the genome taxonomy database. *Bioinformatics* **36**, 1925–1927 (2020).
6. Youngblut, N. D. *et al.* Large-Scale Metagenome Assembly Reveals Novel Animal-Associated Microbial Genomes, Biosynthetic Gene Clusters, and Other Genetic Diversity. *mSystems* **5**, 1–15 (2020).
7. Gilroy, R., Leng, J., Ravi, A., Adriaenssens, E. M. & Oren, A. Metagenomic investigation of the equine faecal microbiome reveals extensive taxonomic diversity. *PeerJ* **10**, e13084 (2022).

SUPPLEMENTARY FIGURES

Supplementary Figure 1 - Description of the first gut microbial gene catalog in horses: contribution of sequencing depth and sample sources to the gene content

(a) Total contig length (Gb) for each sample; (b) Rarefaction curve showing the number of genes from the total gene catalog that could be discovered with increasing numbers of raw sequencing reads. A rarefaction curve for each individual is depicted; (c) Association of predicted gene numbers with the sequencing depth (Gb); (d) Number of predicted genes per sample; (e) The proportions of genes having high abundance (≥ average abundance) in the corresponding samples that they came from

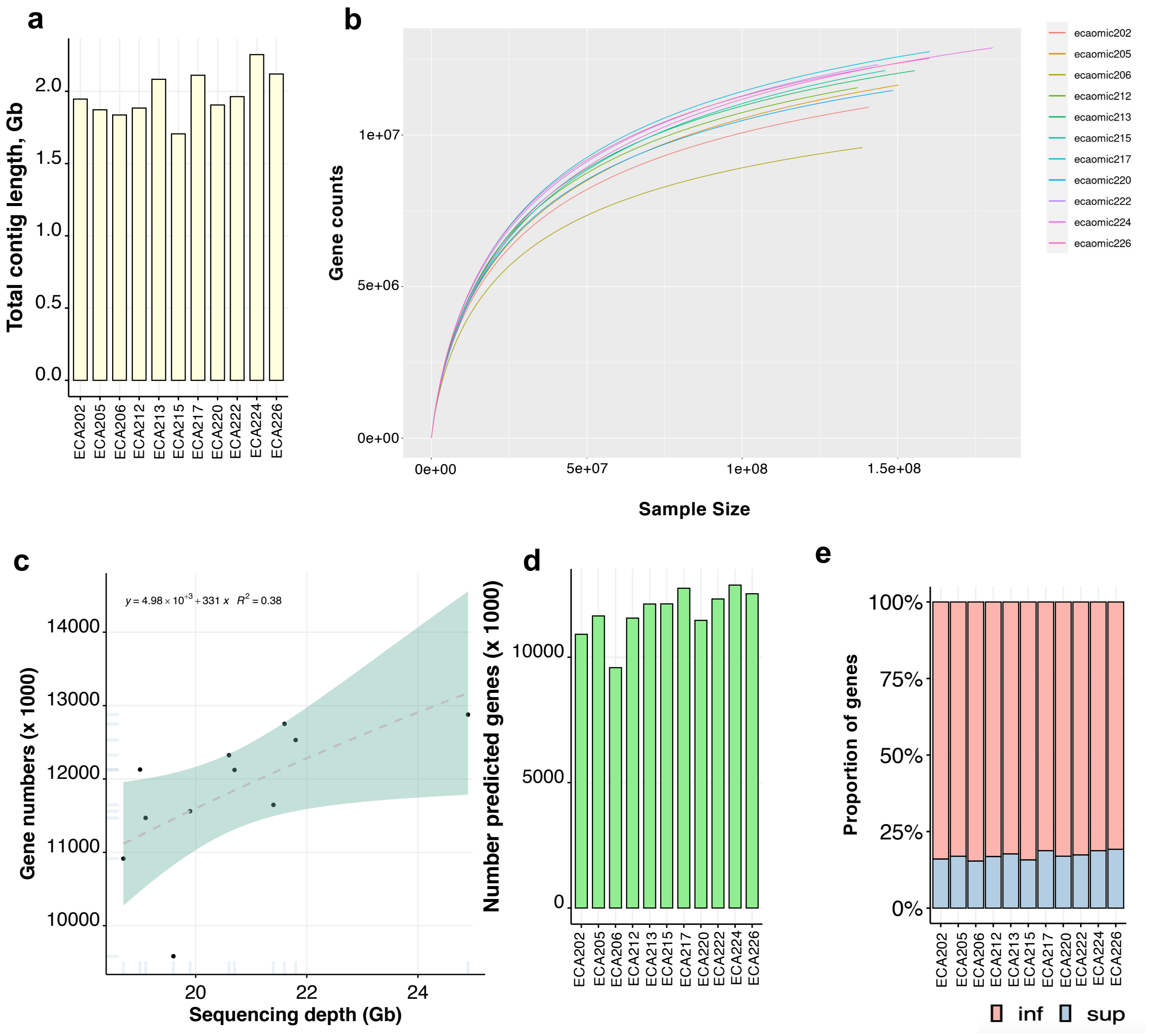

Supplementary Figure 2 - Characteristics of the 372 metagenome-assembled genomes (MAGs)

(a-b) Distribution of completeness and contamination quality metrics, respectively. The 372 MAGs are overlaid on each boxplot, which shows the median, 25th, and 75th percentiles. The whiskers indicate the minima and maxima, and the points lying outside the whiskers of boxplots represent the outliers; (c) Relationship between MAG completeness and contamination percentages. Dot size is proportional to the strain heterogeneity, and it is colored according to the phylum; (d) Relationship regression between MAG size (Kb) and percentage of completeness; (e) Frequency of the contig length (Kb); (f-g) Distribution of the number of N50 value (Kb) and contigs, respectively. The 372 MAGs are overlaid on each boxplot, which shows the median, 25th, and 75th percentiles. The whiskers indicate the minima and maxima, and the points lying outside the whiskers of boxplots represent the outliers; (h) Top 15 MAGs abundance across samples (left) alongside the overlaid abundance histogram and boxplot to describe their distribution in the cohort.

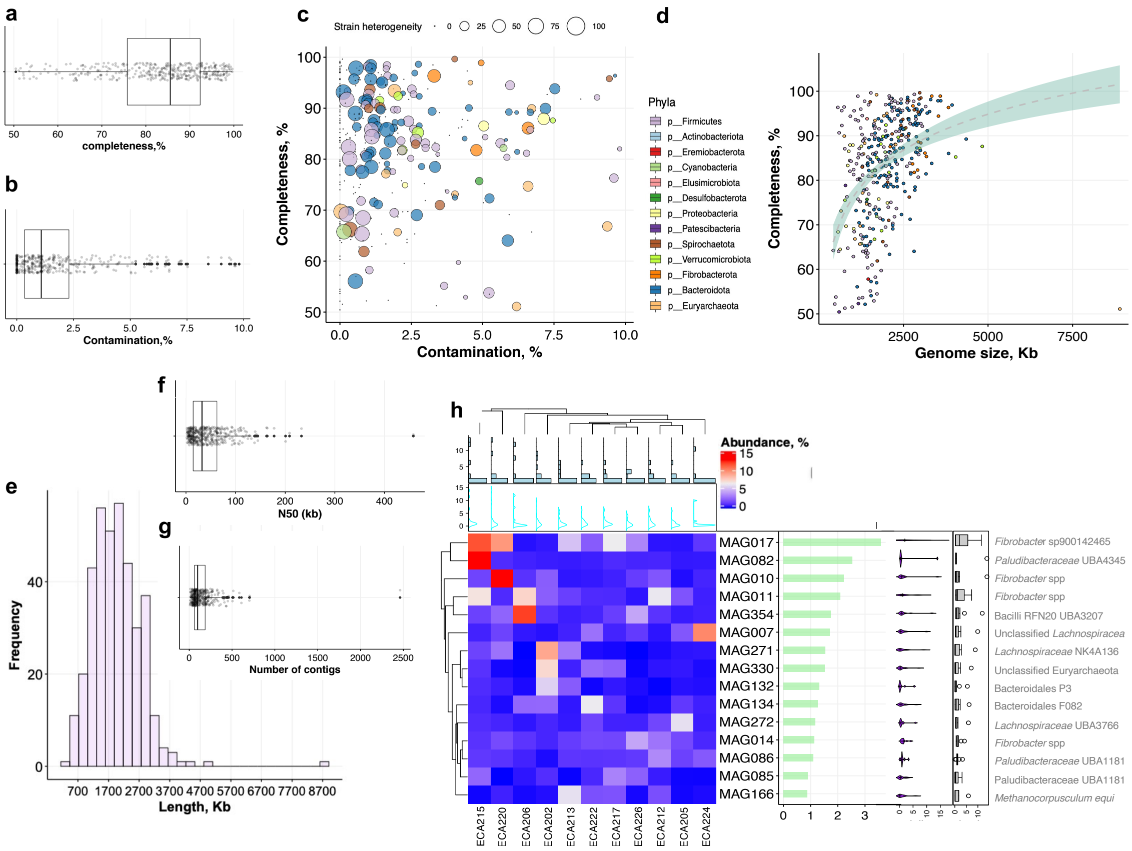

Supplementary Figure 3 – Distribution of the CAZymes families across MAGs
Phylogenetic tree of the MAGs. Each MAG is colored according to its phylum. CAZymes abundance data was integrated into the phylogenetic tree context. CAZymes are colored by family.

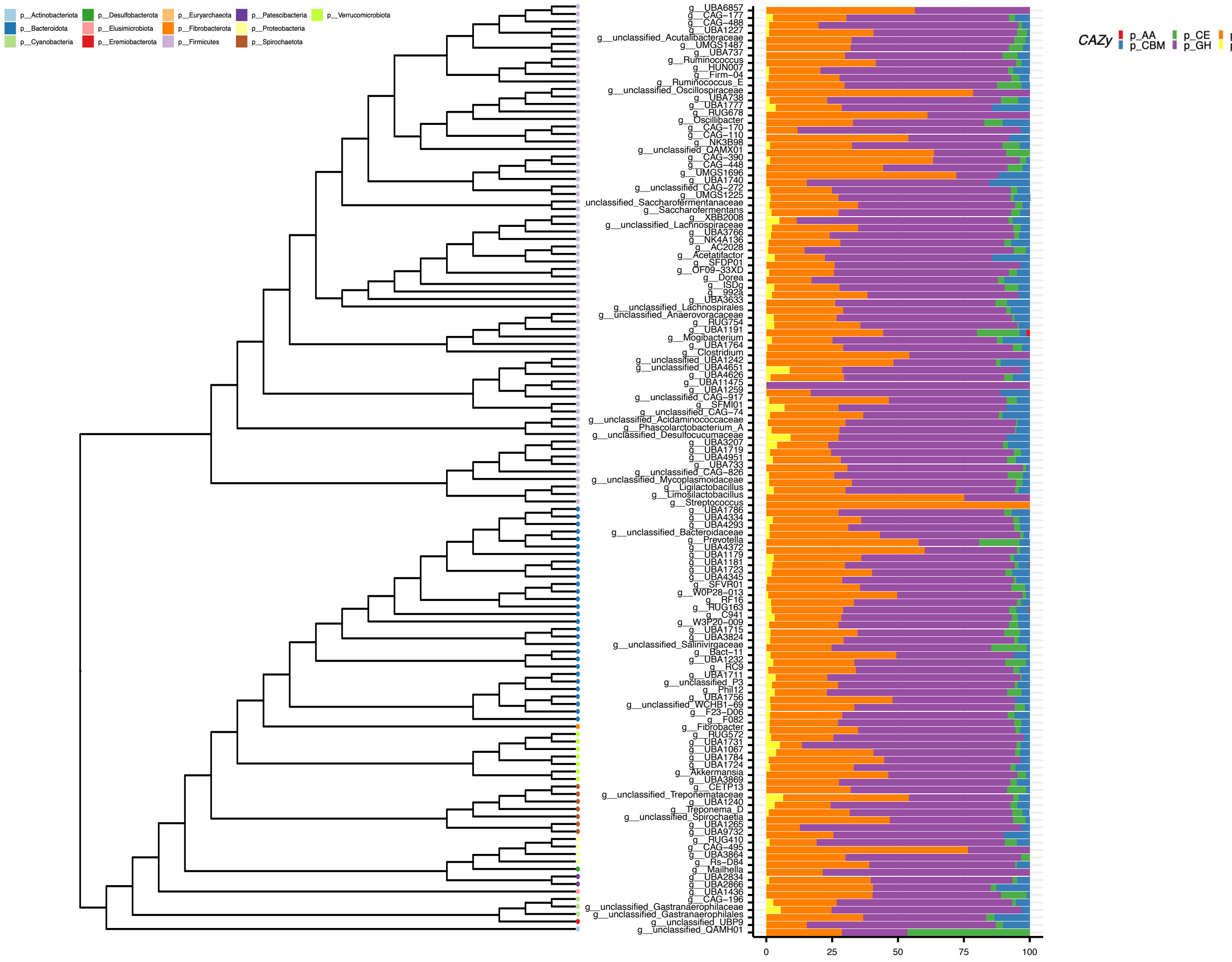

Supplementary Figure 4 - Interconnection between cardiovascular fitness, microbiome composition, and functionality

(a-b) Line plots showing the relationship between frequency (*y*-axis) and the relative abundance (*x*-axis) of the 318 upregulated genera in cluster 1 compared to cluster 2, averaged across cluster 1 (a) and cluster 2 (b); (c) Dot plot representation of log-transformed fold change of dominant phylotypes significantly differed between the individuals with reduced and improved cardiovascular capacity. The logs of fold changes above 0 indicate that phylotypes were more abundant in individuals with reduced cardiovascular capacity than those with improved cardiovascular fitness. By contrast, the negative logs of fold changes indicate that the phylotype abundance was lower in individuals with reduced cardiovascular capacity than in more fit participants. Dots are colored by phylum; (d) Dot plot representation of log-transformed fold change of CAZymes significantly differed between individuals with reduced and improved cardiovascular capacity. The logs of fold changes between 0 and 1.5 indicate that CAZymes were more abundant in less fit individuals. By contrast, the logs of fold changes between 0 and -1.5 show that the CAZymes abundance was lower in individuals with reduced cardiovascular capacity than in more fit individuals. Dots are colored by family.

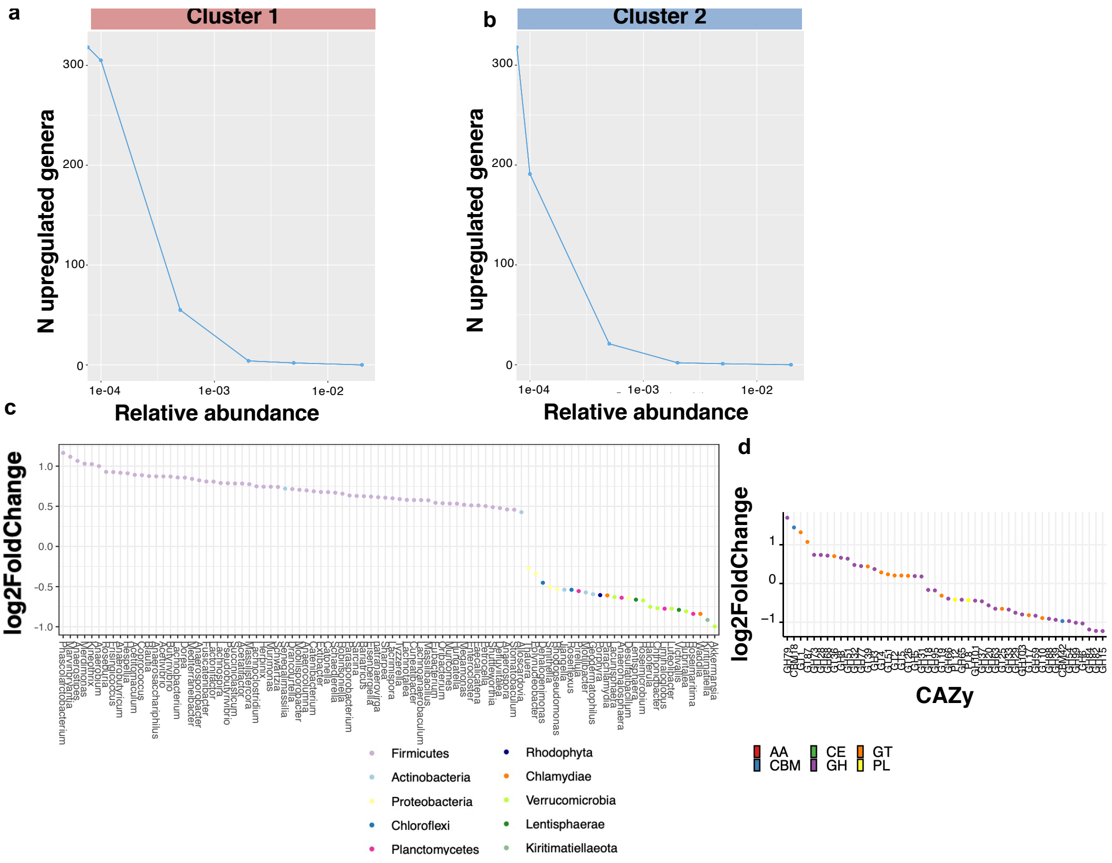

Supplementary Figure 5 - Landscape of gut metagenome in athletes: two meta-communities with different microbial diversity and distinct metagenomic functions

(a-b) PCoA ordination analysis (Bray-Curtis distance) of the CAZymes and KOs profiles estimated from the gut gene catalog, respectively. In all cases, colors indicate community classification: cluster1 (red color) and cluster 2 (blue color); (c) Violin plot representing the percentage of host reads in the raw paired-end reads, according to the clustering groups, respectively. In all cases, colors indicate community classification: cluster1 (red color) and cluster 2 (blue color). Boxplots show the median, 25th, and 75th percentile, the whiskers indicate the minima and maxima, and the points lying outside the whiskers of boxplots represent the outliers. Adjusted *p* values from two-sided Wilcoxon rank-sum test

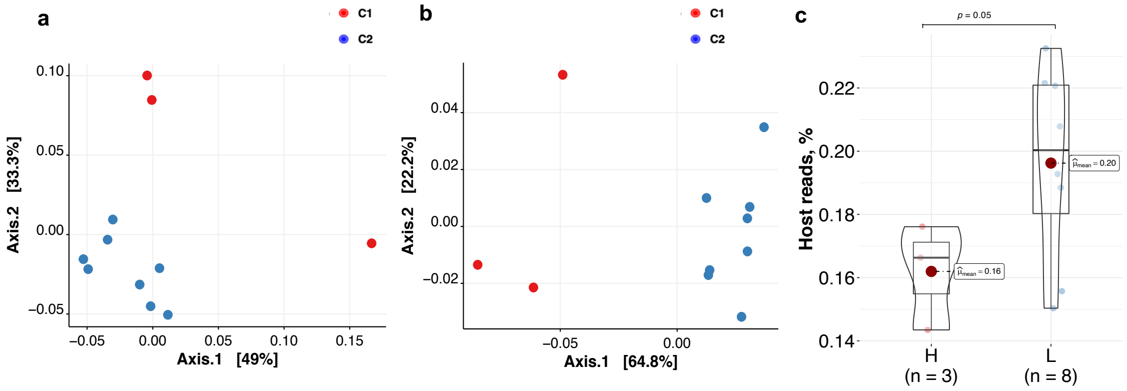

Supplementary Figure 6 - The metagenome-assembled genomes do not capture the functional complexity of the natural population

(a-b) Principal coordinate analysis (PCoA) ordination analysis (Bray-Curtis distance) of CAzymes and KOs estimated from MAGs, respectively; (c) Dot plot representation of log-transformed fold change of MAGs that were significantly different between the individuals with low and high cardiovascular capacity. The logs of fold changes between 0 and 6 indicate that MAGs were more abundant in individuals with lower cardiovascular fitness than high. By contrast, the logs of fold changes between 0 and -6 indicate that the MAG abundance was lower in less fit participants than in more fit horses. Dots are colored by phyla; (d) Distribution of the fraction of CAZymes abundance estimated from MAGs in each sample. CAZymes are colored according to their family.

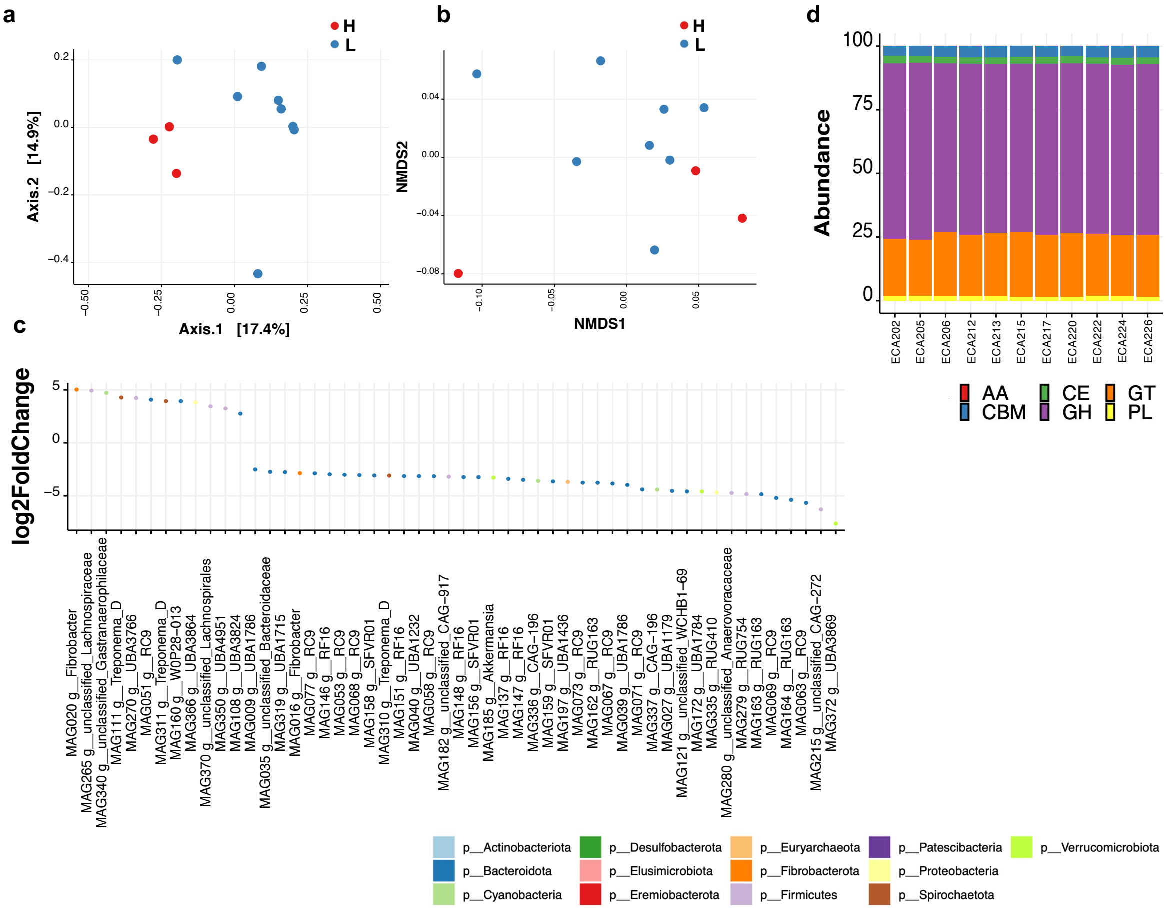

Supplementary Figure 7 – The compositional and functional differences between microbial clusters were not driven by nutrient intake or horse-centered parameters. (a-g) Non-metric multidimensional scaling (NMDS) ordination plot with Bray-Curtis distance and based on the dominant phylotypes. Samples are colored by the intake of daily fiber, hydrolyzable carbohydrates, protein, and fat, sex, age (years), and breed, respectively.

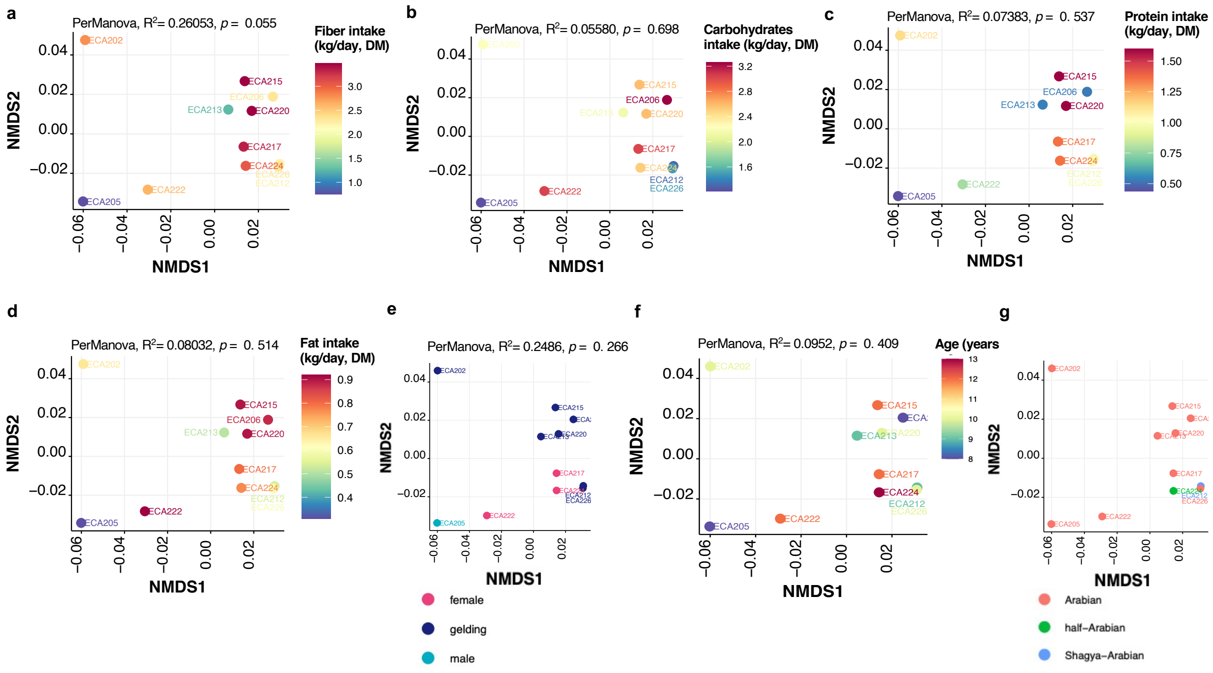

Supplementary Figure 8 - Genetic relationship of the 11 endurance horses in the experiment

(a) Illustration of a six-generation pedigree plot, with different shapes for male (squares) and female (circles); (b) Heatmap of the kinship coefficient matrix, which assesses the genetic resemblance between horses. Each entry in the matrix is the kinship coefficient between two subjects. Animals are arranged in the order of their genetic relatedness. Genetically similar animals are near each other. In the heatmap, red = high values of genetic relatedness, and white = low values of genetic relatedness

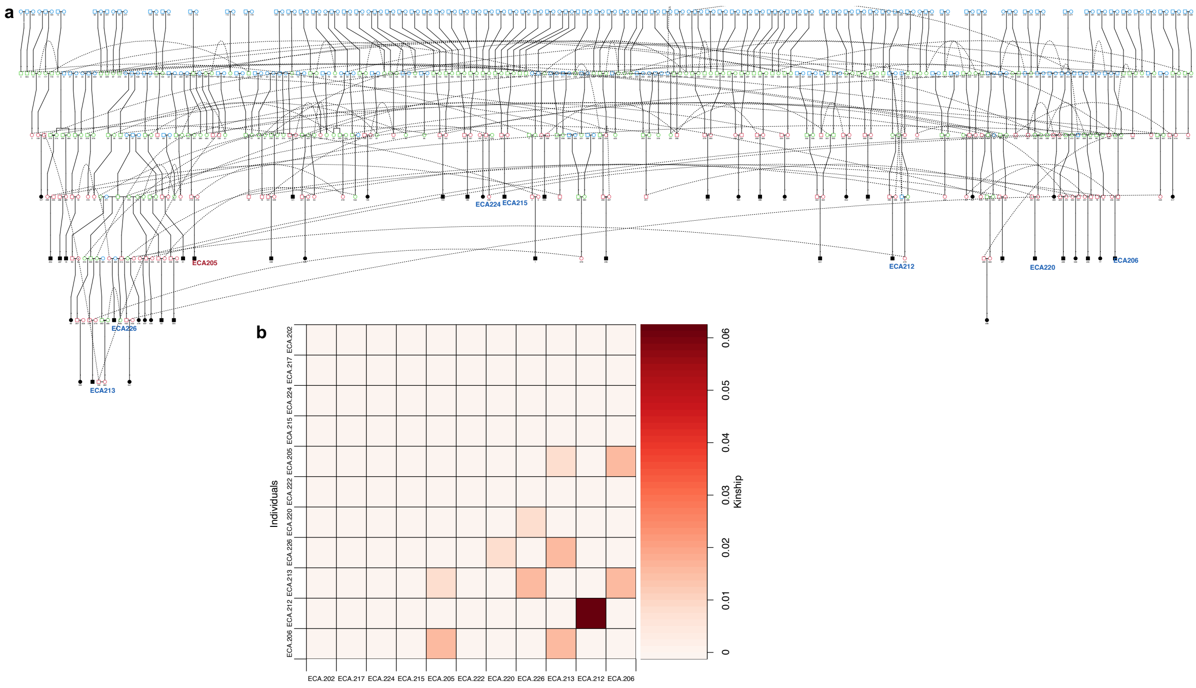

Supplementary Figure 9 – Validation set with 22 independent elite athletes: effect of cardiovascular fitness on the gut microbiome based on 16S rRNA amplicon sequencing
(a) Violin plot representation of the composite of cardiovascular capacity in the validation set: “L” corresponds to “low,” M” to “Medium,” and “H” to “high.” The composite of the cardiovascular capacity was made by combining the post-exercise heart rate, the cardiac recovery time, and the average speed during the race. Adjusted *p* values from two-sided Wilcoxon rank-sum test; (b) PCoA ordination analysis (Bray-Curtis distance) of the relative abundances estimated with the 16S rRNA sequencing in the validation set. Points denote individual samples (n = 22), which are colored according to the clustering group: H (red), M (yellow), and L (blue); (c) Bray-Curtis distance to the centroid of the gut microbial ASVs estimated by the 16S rRNA gene between the L, M and H groups. Adjusted *p* values from Tukey’s Honest significant differences tests; (d) Multivariate sparse partial least-squares discriminant analysis (sPLS-DA) loading plot showing the contributing genera towards the separation between individuals with better cardiovascular fitness (red color) and less fit individuals (blue color). Bar length indicates loading coefficient weight of selected genus, ranked by importance, bottom to top; (e-f) Boxplots of *Ruminococcus* phylotypes and *Dorea* relative abundance, which were significantly different between individuals with better cardiovascular fitness (red color) and less fit individuals (blue color) at the basal time (DESeq2, adjusted p-values < 0.05). In all cases, boxplots show the median, 25th, and 75th percentile, the whiskers indicate the minima and maxima, and the points lying outside the whiskers of boxplots represent the outliers. The box color indicates the group: H (red), M (yellow), and L (blue)

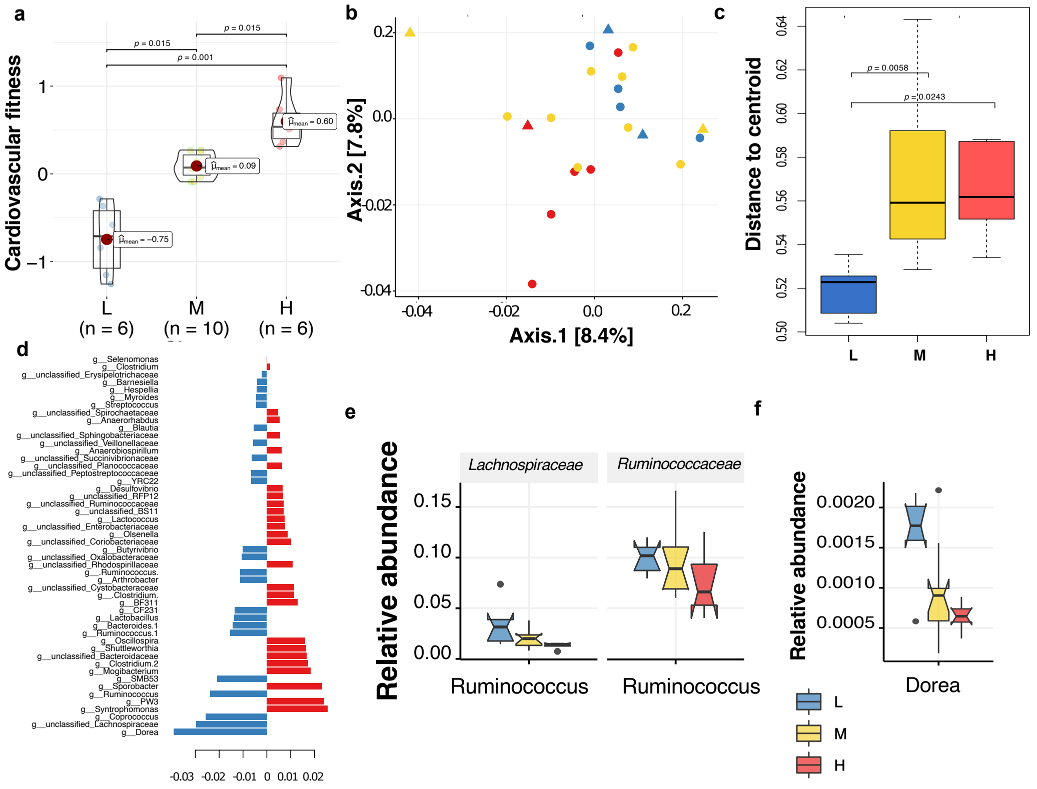

Supplementary Figure 10 - Interconnection between cardiovascular fitness and gut microbiome composition and functionality

(a-c) DIABLO sample plot demonstrating discrimination between groups based on dominant phylotypes data, mitochondrial-related genes expression, and carbohydrate-active enzymes (CAZy) data, respectively; (d) Gut microorganism loads contributing to separation along with component 1 of DIABLO sample plot; (e) Mitochondrial-related genes contributing to separation along with component 1 of DIABLO sample plot. In all cases, bar length indicates the loading coefficient weight of the selected feature, ranked by importance, bottom to top, and colors indicate sample classification, the individuals with high cardiovascular fitness (red color) and the individuals with low cardiovascular fitness (blue color)

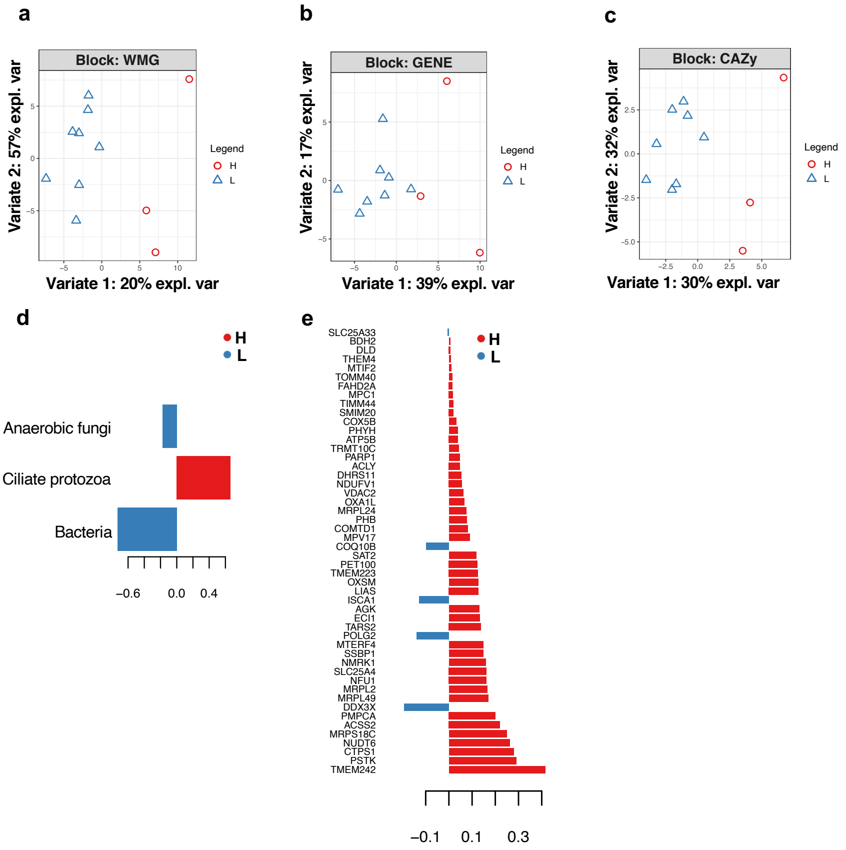

Supplementary Figure 11 – Identification of 16S rRNA contamination data

The frequency distribution of each ASV was calculated as a function of the input DNA concentration using the decontam R package. In this plot, the dashed black line shows the model of a noncontaminant sequence feature for which frequency is expected to be independent of the input DNA concentration. The red line shows the model of a contaminant sequence feature. For a contaminant, the frequency is expected to be inversely proportional to input DNA concentration, as contaminating DNA will make up a more significant fraction of the total DNA in samples with very little total DNA

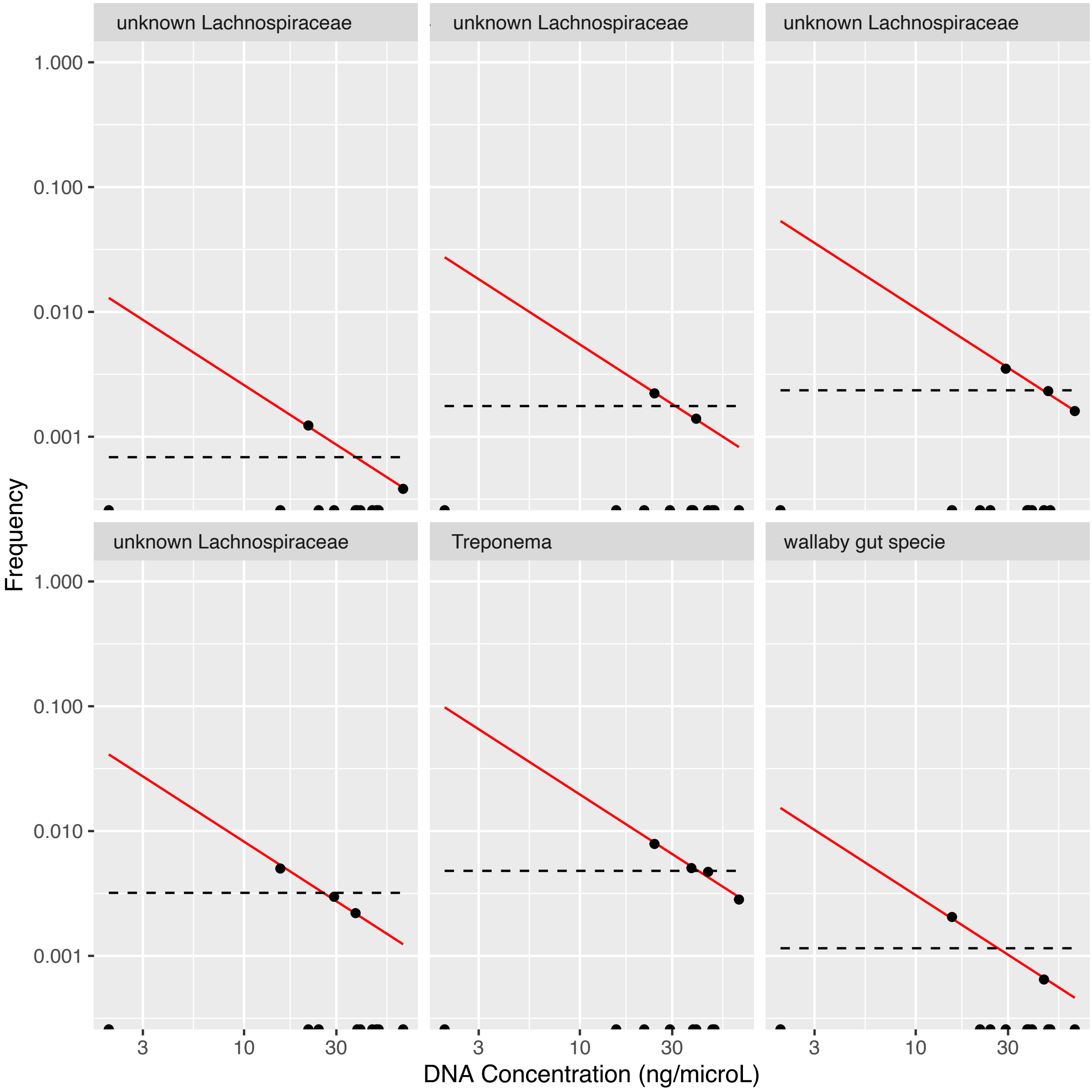

Supplement: Supplementary file 2 — Supplementary Informatio﻿n [file 42003_2022_3977_MOESM2_ESM.pdf]
