# Supplementary material for: Mining the equine gut metagenome: poorly-characterized taxa associated with cardiovascular fitness in endurance athletes
Source: Commun Biol. 2022 Oct 3;5:1032. doi: 10.1038/s42003-022-03977-7 (PMC9529974; doi:10.1038/s42003-022-03977-7)
Supplement: Supplementary file 3 — Description of Additional Supplementary Files [file 42003_2022_3977_MOESM3_ESM.pdf]

# Description of Additional Supplementary Files

**File name:** Supplementary Data 1

**Description:** Metadata of horses recruited in the experiment

**File name:** Supplementary Data 2

**Description:** Sequencing data and assembly statistics for each sample and quality assessment for the gut microbiome gene catalog

**File name:** Supplementary Data 3

**Description:** Gene counts of each phylotype detected in the equine gut microbiome gene catalog. Taxonomic profiling was done with Kaiju

**File name:** Supplementary Data 4

**Description:** Gene counts of each phylotype detected in the equine core gene catalog. Taxonomic profiling was done with Kaiju

**File name:** Supplementary Data 5

**Description:** Gene counts of each KO detected from the equine gut microbiome gene catalog

**File name:** Supplementary Data 6

**Description:** Gene counts of each CAZymes detected from the equine gut microbiome gene catalog and their estimated mechanisms

**File name:** Supplementary Data 7

**Description:** List of acquired antimicrobial resistance (AMR) genes observed in each horse

**File name:** Supplementary Data 8

**Description:** Assembly statistics of the 372 metagenome-assembled genomes (MAGs), abundance, and distributions of CAZymes and KOs

**File name:** Supplementary Data 9

**Description:** Top 25% of most dominant phylotypes sorted by their abundance and ubiquitousness found in more than half of the samples. Taxonomic profiling was done with Kaiju. For each individual, the phylotype taxonomic assignment and their counts are depicted

**File name:** Supplementary Data 10

**Description:** Abundance of genera detected by 16S rRNA gene sequencing in each sample

**File name:** Supplementary Data 11

**Description:** Number of ASVs and relative abundance of genera detected by 16S rRNA gene sequencing

**File name:** Supplementary Data 12

**Description:** Comparison of differential abundance of dominant microbial

phylotypes between individuals from different clusters. Differences were calculated between the two groups based on the DESeq2 model followed by Benjamini and Hochberg multiple test correction. The table shows the phylotypes that significantly varied in abundance between cluster 2 and cluster 1 individuals. The information relative to the log fold change and the adjusted p-value is provided

**File name:** Supplementary Data 13

**Description:** Comparison of differential abundance of CAZymes between individuals from cluster 1 and cluster 2. Differences were calculated between the two groups based on the DESeq2 model followed by Benjamini and Hochberg multiple test correction. The table shows the CAZYmes that significantly varied in abundance between cluster 2 and cluster 1 individuals. The information relative to the log fold change and the adjusted p-value is provided

**File name:** Supplementary Data 14

**Description:** Comparison of differential abundance of KOs between individuals from cluster 1 and cluster 2. Differences were calculated between the two groups based on the DESeq2 model followed by Benjamini and Hochberg multiple test correction. The table shows the CAZYmes that significantly varied in abundance between cluster 2 and cluster 1 individuals. The information relative to the log fold change and the adjusted p-value is provided. Only KOs related to metabolism are described.

**File name:** Supplementary Data 15

**Description:** List of differentially expressed mitochondrial-related genes. Gene description, gene localizations, and gene types were determined according to IPA (https://www.qiagenbioinformatics.com/products/ingenuity-pathway-analysis). Columns from I to S show the expression values obtained by subtracting the T0 (baseline) from the T1 (post-exercise) expression matrix values, i.e., by calculating the ratio between T1 and T0 log scaled expression values from the two matrices

**File name:** Supplementary Data 16

**Description:** Relative abundance of metabolites pre-and post-exercise measured in serum from 11 horses under the study

**File name:** Supplementary Data 17

**Description:** Biochemical parameters pre-and post-exercise measured in serum from 11 horses under the study

**File name:** Supplementary Data 18

**Description:** Acylcarnitine values (mmol/L ± standard deviation) pre-and post-exercise measured in serum from 11 horses under the study

**File name:** Supplementary Data 19

**Description:** Summary of the significant correlations between environmental and host variables and microbial beta-diversity ordination on NMDS plots. +/- designates the direction of the association between the fit variable and the NMDS axis. Squared correlation coefficients are depicted as estimators of the goodness of fit and the empirical p-values for each variable

**File name:** Supplementary Data 20

**Description:** Metadata of the horses recruited in the validation cohort

**File name:** Supplementary Data 21

**Description:** ASV taxonomic assignments and ASV counts for the 22 horses in the validation set based on 16S rRNA sequencing

**File name:** Supplementary Data 22

**Description:** Fecal pH and fecal short-chain fatty acids measurements in the 11 horses under the study before the endurance race, as well as their concentrations of bacteria, protozoa, and anaerobic fungi in the feces

**File name:** Supplementary Data 23

**Description:** The source data underlying the graphs and charts. This table contains the ASV matrix and the ASV annotation. The corresponding metadata is depicted in Supplementary Data 1
