# Supplementary material for: Mining the equine gut metagenome: poorly-characterized taxa associated with cardiovascular fitness in endurance athletes
Source: Commun Biol. 2022 Oct 3;5:1032. doi: 10.1038/s42003-022-03977-7 (PMC9529974; doi:10.1038/s42003-022-03977-7)
Supplement: Supplementary file 27 — Reporting Summary [file 42003_2022_3977_MOESM27_ESM.pdf]

nature portfolio

Corresponding author(s): Núria Mach

Last updated by author(s): Jul 24, 2022

Reporting Summary

Nature Portfolio wishes to improve the reproducibility of the work that we publish. This form provides structure for consistency and transparency in reporting. For further information on Nature Portfolio policies, see our Editorial Policies and the Editorial Policy Checklist.

Statistics

For all statistical analyses, confirm that the following items are present in the figure legend, table legend, main text, or Methods section.

n/a	Confirmed	
☐	☒	The exact sample size (*n*) for each experimental group/condition, given as a discrete number and unit of measurement
☐	☒	A statement on whether measurements were taken from distinct samples or whether the same sample was measured repeatedly
☐	☒	The statistical test(s) used AND whether they are one- or two-sided<br>*Only common tests should be described solely by name; describe more complex techniques in the Methods section.*
☐	☒	A description of all covariates tested
☐	☒	A description of any assumptions or corrections, such as tests of normality and adjustment for multiple comparisons
☐	☒	A full description of the statistical parameters including central tendency (e.g. means) or other basic estimates (e.g. regression coefficient) AND variation (e.g. standard deviation) or associated estimates of uncertainty (e.g. confidence intervals)
☐	☒	For null hypothesis testing, the test statistic (e.g. *F*, *t*, *r*) with confidence intervals, effect sizes, degrees of freedom and *P* value noted<br>*Give P values as exact values whenever suitable.*
☒	☐	For Bayesian analysis, information on the choice of priors and Markov chain Monte Carlo settings
☒	☐	For hierarchical and complex designs, identification of the appropriate level for tests and full reporting of outcomes
☒	☐	Estimates of effect sizes (e.g. Cohen's *d*, Pearson's *r*), indicating how they were calculated

Our web collection on statistics for biologists contains articles on many of the points above.

Software and code

Policy information about availability of computer code

Data collection

No software was used for data collection

Data analysis

Phylogenic analysis based on the 16S rRNA sequencing data was performed using the Divisive Amplicon Denoising Algorithm (DADA2) plug-in for QIIME 2 (v.2021.2). Taxonomic assignments of amplicon sequence variant (ASV) were done by importing Greengenes 16S rRNA Database (release 13.8). The phyloseq (v.1.36.0), vegan (v.2.5.7) and microbiome (v.1.14.0) packages were used in R (v.4.1.0) for the downstream steps of analysis.

The establishment and assessment of the quality and representation of the microbiome gene catalog and metagenome-assembled genomes (MAGs) was performed through the metagenomic ATLAS pipeline (v.2.4.4). The ATLAS configuration file with all settings for the pipeline are available at the INRAE data repository (https://data.inrae.fr/dataset.xhtml?persistentId=doi:10.15454/NGBSPC). Using tools from the BBmap suite (v.37.99), reads were quality trimmed and contamination from the horse genome were filtered out (available at NCBI sequence archive with the accession number GCA_002863925.1; Equus_caballus.EquCab3.0). Reads were error corrected and merged before assembly with metaSPAdes (v.3.13.1). QUAST (v.5.0.2) was used to evaluate the quality of each sample assembly. Contigs from single samples were binned using MetaBAT 2 (v.2.14) and Maxbin 2.0 (v.2.2.7) and their predictions were combined using DAS Tool (v.1.1.2-1).
The quality of MAGs was then assessed using checkM (v.1.1.3). The dRep (v.2.2.2) was used to obtain a non-redundant set of MAGs. The taxonomic annotation was performed through Prodigal (v.2.6.3), according to the genome taxonomy database (GTDB-tk) release 95, v.5.0 (July 17, 2020). MAG phylogenetic trees were built based on markers from GTDB-Tk and CheckM and visualized using ggtree (v.3.0.2) in R package.

The predicted gene and proteins extracted by Prodigal during the CheckM pipeline were compared to the EggNOG database 5.0 using eggnog-mapper (v2.0.1). From this output, KEGG annotation (Kyoto Encyclopedia of Genes and Genomes) and CAZymes annotation (Carbohydrate-active Enzyme) were extracted. Pathways attributed to each KO were annotated from the KEGG database (downloaded 23-October-2021;

https://www.genome.jp/brite/ko00001). The same software were used for the gene catalog prediction and taxonomic and functional annotation.
The taxonomy of high-quality clean paired reads was performed using kaiju (v.1.8.0) and the NCBI nr reference database (released on May 25th 2020).
For the resistome analysis, the high-quality clean paired reads were aligned to the ResFinder database (accessed March 2018, v.4.0) using bowtie2 (v.2.3.5).

Biodiversity and richness analysis, the inference and analysis of SPIEC-EASI microbiome networks and the integrative statistical analysis were conducted in R (v.4.1.0) using phyloseq (v.1.36.0), vegan (v.2.5.7), microbiome (v.1.14.0), SpiecEasi (v.1.1.1), igraph (v.1.2.6), DIABLO of the mixOmics R package (http://mixomics.org/, v6.12.2), DESeq2 (v.1.32.0) packages and Cytoscape (v.3.8.2).

For manuscripts utilizing custom algorithms or software that are central to the research but not yet described in published literature, software must be made available to editors and reviewers. We strongly encourage code deposition in a community repository (e.g. GitHub). See the Nature Portfolio guidelines for submitting code & software for further information.

Data

Policy information about availability of data

All manuscripts must include a data availability statement. This statement should provide the following information, where applicable:
- Accession codes, unique identifiers, or web links for publicly available datasets
- A description of any restrictions on data availability
- For clinical datasets or third party data, please ensure that the statement adheres to our policy

Microarray expression data (MIAME compliant) are available in Gene Expression Omnibus (GEO) repository under the accession number GSE163767 (https://www.ncbi.nlm.nih.gov/geo/query/acc.cgi?acc=GSE163767).

Metabolomic data are available in the NIH Common Fund's Data Repository and Coordinating Center UrqK1489; (http://dev.metabolomicsworkbench.org:22222/data/DRCCMetadata.php?Mode=Study&StudyID=ST000945).

The gut metagenome 16S rRNA targeted locus data are available in the DDBJ/EMBL/GenBank under the BioProject PRJNA438436. The accession numbers of the BioSamples included here are SAMN08715729, SAMN08715728, SAMN08715727, SAMN08715725, SAMN08715723, SAMN08715721, SAMN08715719, SAMN08715718, SAMN08715714, SAMN08715713, SAMN08715710. The SRR accession numbers for the 16S rRNA targeted locus data are: SRR13664931, SRR13664928, SRR13664927, SRR13664925, SRR13664924, SRR13664923, SRR13664921, SRR13664919, SRR13664918, SRR13664917 and SRR13664916, respectively.

Moreover, the raw metagenomic sequence data of the 11 athletes have been deposited at DDBJ/ENA/GenBank Whole Genome Shotgun under the same BioProject ID PRJNA438436 and BioSamples numbers. The accession numbers ranged from SRR17543914 to SRR17543904.

All metagenome assemblies and sequences of MAGs have also been deposited at DDBJ/ENA/GenBank Whole Genome Shotgun under the same BioProject ID PRJNA438436, with the genome accession numbers ranging from JAKSHS000000000 to JAKSVZ000000000. They are also available at the INRAE institutional data repository powered by Dataverse with DOI: www.doi.org/10.15454/NGBSPC 92.

Lastly, the horse gut microbiome gene catalog is available at DDBJ/ENA/GenBank under the same BioProject ID PRJNA438436 and the accession numbers from JALNLV000000000 to JALNLY000000000. The catalog is also available at the INRAE institutional data repository powered by Dataverse with DOI: www.doi.org/10.15454/NGBSPC 92.

Datasets generated or analyzed during the study are included in this published article as Supplementary Data. Other data supporting this study's findings are available in the INRAE institutional data repository powered by Dataverse with DOI: www.doi.org/10.15454/NGBSPC 92. They have been appropriately specified in the text where required.

The source data underlying the graphs and charts presented in the main figures are stored in Supplementary Data 23. It contains a single experiment-level phyloseq object with the ASV matrix, all related phylogenetic sequencing data, annotation, and metadata.

Data sets and products generated from the raw sequence data are available at the INRAE institutional data repository powered by Dataverse with DOI: www.doi.org/10.15454/NGBSPC.
(https://data.inrae.fr/dataset.xhtml?persistentId=doi:10.15454/NGBSPC). They have been appropriately specified in the text where required. All other data is available in the Supplementary Data.

The following databases can be accessed using the following links: CAZy (http://www.cazy.org/) (CAZyDB.07312018), GTDB-Tk (https://github.com/Ecogenomics/GTDBTk)(release 95, v.5.0), KEGG (https://www.kegg.jp/kegg/download/) (downloaded 23-October-2021), NCBI nr (https:// www.ncbi.nlm.nih.gov/ (release May 25th 2020), Greengenes 16S rRNA Database (https://greengenes.secondgenome.com (release 13.8).

Field-specific reporting

Please select the one below that is the best fit for your research. If you are not sure, read the appropriate sections before making your selection.

☐ Life sciences ☐ Behavioural & social sciences ☒ Ecological, evolutionary & environmental sciences

For a reference copy of the document with all sections, see nature.com/documents/nr-reporting-summary-flat.pdf

Ecological, evolutionary & environmental sciences study design

All studies must disclose on these points even when the disclosure is negative.

Study description	Elite endurance horses were used as in vivo model system for characterizing the relationships between gut microbiome and endurance performance while controlling for known confounding factors. To understand if and how gut microbiome functions are responsible for better adaptations to fatigue resistance, as well as success in athletic performances we first built a gene catalog of the equine gut microbiome and a repertoire of metagenome-assembled genomes (MAGs). Then after, we used the holo-omic approach that incorporates multi-omic data from host and microbiome domains to decipher how gut microbiome contributes to exercise performance.
Research sample	11 elite horses in the experimetal set and 22 elite horses in the validation set
Sampling strategy	Endurance horses were recruited under field conditions on a voluntary bases. Unfortunatelly, a high percentage of owners invited to participate in the study were worried about the mechanical or chemical phlebitis after blood sampling (especially before the endurance event) and thus declined the invitation before the ride.
Data collection	Data collected in the field was manually recorded in Excel tables
Timing and spatial scale	All the participants enrolled in the study (experimental and validation set) competed in the same event during October 2015 in Fontainebleau (France). During the race, the weather conditions, terrain difficulty and altitude were the same for all the participants enrolled in the study. The average air temperature was 15°C, with a maximum of 20°C and a minimum of 11°C, the average air humidity was 88%, and no rain was recorded.
Data exclusions	Animals were not included if they presented gastrointestinal disorders during the four months prior to enrollment, if they were treated with antibiotic during the four months prior to enrollment or treated with anthelmintic medication within 60 days before the race
Reproducibility	The association between the cardiovascular fitness and the gut microbiome composition found in the 11 elite horses based on their their metagenome data was confirmed by the 16S rRNA sequence data from the gut microbiota of 22 independent highly trained endurance horses. For instance, taxa such as Barnesiella, Blautia, Butyrivibrio, Coprococcus, Dorea, Desulfovibrio, Hespellia, Lachnospira, Myroides and L-Ruminococcus were commonly found in less fit athletes in both discovery and validation sets. Contrastingly, individuals with improved cardiovascular fitness harbored a multitude of minor players, as observed in the discovery set. These data mainly confirm the negative association between Firmicutes (notably Ruminococcus and Dorea taxa) and cardiovascular fitness.
Randomization	Individuals were not randomly selected.
Blinding	Blinding is not necessary for this study as we do not study human subjects.

Did the study involve field work? ☒ Yes ☐ No

Field work, collection and transport

Field conditions	Pretreatment of the blood and feces samples was carried out immediately after the collection because the access to refrigeration and electrical power supply was available under the field conditions, as well as a tank filled with liquid nitrogen.<br>For metabolome profiling, whole blood samples were collected from each horse before and after the race in sodium fluoride and oxalate tubes in order to inhibit further glycolysis that may increase the lactate levels after sampling. Whole blood drawn for plasma generation was put at once at 4ºC to minimize the metabolic activity of cells and enzymes and kept the metabolite pattern almost stable. Clotting time at 4ºC was strictly controlled for all samples to avoid cell lyses that affect the components of the metabolome. After clotting at 4ºC, the plasma was separated from the blood cells and subsequently transported to the lab at 4ºC and frozen at -80 °C (no more than 5 h later, in all cases). For blood biochemical assays, blood samples were collected in 10 mL BD Vacutainer EDTA tubes before and at the end of the endurance event. After clotting, the tubes were centrifuged and the harvested serum was stored at 4°C until analysis (no more than 48h later, in all cases). Similarly, blood samples were collected in plain tubes prior to and within 30 minutes of the end of the ride for the acylcarnitine profiling. After clotting, the tubes were centrifuged and the harvested serum was stored at 4 °C for no more than 5h and subsequently stored at -80 °C.<br>For transcriptome profiling, whole blood was collected into PaxGene Blood RNA tubes because minimal handling is required and the latter tubes can (according to the manufacturer's documentation) be stored for up to three days at room temperature. Tubes were stored at room temperature no more than 5h in all cases and subsequently stored at -80 °C.<br>Feces samples aliquots for SCFA analysis and DNA extraction were snap-frozen in the field.
Location	Fontainebleau, France. Fontainebleau is 150m above sea level (48.408; 2.699)
Access & import/export	Permissions granted to the project by the Alfort Veterinary School and the University of Paris Est (reference: 12/07/11-1)
Disturbance	No plant or animal life was disturbed during sampling

Reporting for specific materials, systems and methods

We require information from authors about some types of materials, experimental systems and methods used in many studies. Here, indicate whether each material, system or method listed is relevant to your study. If you are not sure if a list item applies to your research, read the appropriate section before selecting a response.

Materials & experimental systems

n/a	Involved in the study
☒	☐ Antibodies
☒	☐ Eukaryotic cell lines
☒	☐ Palaeontology and archaeology
☐	☒ Animals and other organisms
☒	☐ Human research participants
☒	☐ Clinical data
☒	☐ Dual use research of concern

Methods

n/a	Involved in the study
☒	☐ ChIP-seq
☒	☐ Flow cytometry
☒	☐ MRI-based neuroimaging

Animals and other organisms

Policy information about studies involving animals; ARRIVE guidelines recommended for reporting animal research

Laboratory animals

The study did not involve laboratory animals

Wild animals

The study did not involve wild animals

Field-collected samples

For metabolome profiling, whole blood samples were collected from each horse before and after the race in sodium fluoride and oxalate tubes in order to inhibit further glycolysis that may increase the lactate levels after sampling. Whole blood drawn for plasma generation was put at once at 4ºC to minimize the metabolic activity of cells and enzymes and kept the metabolite pattern almost stable. Clotting time at 4ºC was strictly controlled for all samples to avoid cell lyses that affect the components of the metabolome. After clotting at 4ºC, the plasma was separated from the blood cells and subsequently transported to the lab at 4ºC and frozen at -80 °C (no more than 5 h later, in all cases). For blood biochemical assays, blood samples were collected in 10 mL BD Vacutainer EDTA tubes before and at the end of the endurance event. After clotting, the tubes were centrifuged and the harvested serum was stored at 4°C until analysis (no more than 48h later, in all cases). Similarly, blood samples were collected in plain tubes prior to and within 30 minutes of the end of the ride for the acylcarnitine profiling. After clotting, the tubes were centrifuged and the harvested serum was stored at 4 °C for no more than 5h and subsequently stored at -80 °C.
For transcriptome profiling, whole blood was collected into PaxGene Blood RNA tubes because minimal handling is required and the latter tubes can (according to the manufacturer's documentation) be stored for up to three days at room temperature. Tubes were stored at room temperature no more than 5h in all cases and frozen at -80ºC until analysis.
Feces samples aliquots for SCFA analysis and DNA extraction were snap-frozen in the field. A tank filled with liquid nitrogen was available.

Ethics oversight

The study protocol was reviewed and approved by the local animal care and use committee (ComEth EnvA-Upec-ANSES, reference: 11-0041, dated July 12th 2011) for horse study. All the protocols were conducted in accordance with EEC regulation (no 2010/63/UE) governing the care and use of laboratory animals, which has been effective in France since the 1st of January 2013. In all cases, the owners and riders provided their informed consent prior to the start of sampling procedures with the animals.

Note that full information on the approval of the study protocol must also be provided in the manuscript.
